# Universality of Logarithmic Loss in Fixed-Length Lossy Compression †

**DOI:** 10.3390/e21060580

**Published:** 2019-06-10

**Authors:** Albert No

**Affiliations:** Department of Electronic and Electrical Engineering, Hongik University, Seoul 04066, Korea; albertno@hongik.ac.kr; Tel.: +82-2-320-1649

**Keywords:** categorical data clustering, fixed-length lossy compression, logarithmic loss, rate-distortion

## Abstract

We established a universality of logarithmic loss over a finite alphabet as a distortion criterion in fixed-length lossy compression. For any fixed-length lossy-compression problem under an arbitrary distortion criterion, we show that there is an equivalent lossy-compression problem under logarithmic loss. The equivalence is in the strong sense that we show that finding good schemes in corresponding lossy compression under logarithmic loss is essentially equivalent to finding good schemes in the original problem. This equivalence relation also provides an algebraic structure in the reconstruction alphabet, which allows us to use known techniques in the clustering literature. Furthermore, our result naturally suggests a new clustering algorithm in the categorical data-clustering problem.

## 1. Introduction

Logarithmic loss is a unique distortion measure in the sense that it allows a “soft” estimation (or reconstruction) of the source. Although logarithmic loss plays a crucial role in learning theory, not much work has been published regarding lossy compression until recently. A few exceptions are a line of work on multiterminal source coding [1,2,3], the single-shot approach to lossy source coding under logarithmic loss [4], and several universal properties of logarithmic loss in information theory [5,6,7]. In [4], Shkel and Verdú focused on the lossy-compression problem when the distortion measure is given by logarithmic loss. On the other hand, Jiao et al. justified logarithmic loss by showing it is the only loss function that satisfies a natural data-processing requirement [5]. Painsky and Wornell provided a universal property of logarithmic loss in the context of classification. In [7], No focused on the universal property of logarithmic loss in the successive refinement problem. We would also like to point out that the information bottleneck method [8,9,10,11] is related to lossy compression under logarithmic loss. Indeed, it is equivalent to the noisy lossy-compression problem under logarithmic loss [12].

In this paper, we present a new universal property of logarithmic loss in fixed-length lossy-compression problems. Consider an arbitrary fixed-length lossy-compression problem, where source and reconstruction alphabets 𝒳 and $\hat{X}$ are discrete. Suppose arbitrary distortion measure $d:X\times \hat{X}$ is given. Then, we show that there exists a corresponding fixed-length lossy-compression problem where the source alphabet remains the same, but the reconstruction alphabet is a set of distributions on 𝒳, and the distortion measure is logarithmic loss. This implies that there is a correspondence between any fixed-length lossy-compression problem under an arbitrary distortion measure and that under logarithmic loss. The correspondence is in the following strong sense:

- optimal schemes for the two problems are the same; and
- a good scheme for one problem is also a good scheme for the other.

We are more precise about the “optimal” and “goodness” of the scheme in later sections. This finding essentially implies that it is enough to consider the lossy-compression problem under logarithmic loss.

The above correspondence provides new insights into the fixed-length lossy-compression problem. In general, the reconstruction alphabet in the lossy-compression problem does not have any well-defined operations. However, in the corresponding lossy compression under logarithmic loss, reconstruction symbols are probability distributions that have their own algebraic structure. Thus, under the corresponding setting, we can apply various techniques, such as the information geometric approach, clustering with Bregman divergence, and relaxation of the optimization problem. Furthermore, the equivalence relation suggests a new algorithm in the categorical data-clustering problem, where data are not in the continuous space.

The remainder of the paper is organized as follows. In Section 2, we revisit some of the known results of logarithmic loss and fixed-length lossy compression. Section 3 is dedicated to the equivalence between lossy compression under arbitrary distortion measures and that under logarithmic loss. In Section 4, we present the geometric interpretation of our result. We provide the log-convex relaxation of lossy compression and connection to the clustering problems in Section 5. Finally, we conclude in Section 6.

*Notation*: Uppercase *X* denotes a random variable, where 𝒳 denotes a set of alphabet. On the other hand, lowercase *x* denotes a specific possible realization of random variable *X*, i.e., x∈X. Similarly, $X^{n}$ denotes an *n*-dimensional random vector $\left(X_{1},X_{2},\ldots ,X_{n}\right)$ while lowercase $x^{n}$ denotes a realization of $X^{n}$. The absolute value of function |f| denotes a size of image of function f:X→Y, i.e., |{f(x):x∈X}|. If it was clear from the context, we used $\sum_{x}$ instead of $\sum_{x\in X}$. We used a natural logarithm and nats instead of bits.

## 2. Preliminaries

### 2.1. Logarithmic Loss

Suppose 𝒳 is a finite set of discrete symbols, and M(X) is the set of probability measures on 𝒳. For x∈X and q∈M(X), the definition of logarithmic loss ℓ:X×M(X)[0,∞] is given by

$$\begin{array}{c} \ell \left(x,q\right)=\log\frac{1}{q(x)}. \end{array}$$

### 2.2. Fixed-Length Lossy Compression

In this section, we briefly introduce the basic settings of the fixed-length lossy-compression problem [13]. In a fixed-length lossy-compression setting, we have a source *X* with finite alphabet X={1,…,r} and source distribution $p_{X}$. An encoder f:X→{1,…,M} maps the source symbol to one of *M* messages. On the other side, a decoder $g:\{1,\ldots ,M\}\to \hat{X}$ maps the message to actual reconstruction $\hat{X}$, where the reconstruction alphabet is also finite $\hat{X}=\{1,\ldots ,s\}$. Let $d:X\times \hat{X}\to [0,\infty )$ be a distortion measure between source and reconstruction.

First, we can define the code that the expected distortion is lower than a given distortion level.

**Definition** **1** (Average distortion criterion)**.** *An (M,D) code is a pair of an encoder f with |f|≤M and a decoder g, such that*

$$\begin{array}{c} E[d(X,g(f(X)))]\leq D. \end{array}$$

*The minimum number of codewords required to achieve average distortion not exceeding D is defined by*

$$\begin{array}{c} M^{⋆}\left(D\right)=\min\left\{M:\exists \left(M,D\right)code\right\}. \end{array}$$

*Similarly, we can define the minimum achievable average distortion given number of codewords M.*

$$\begin{array}{c} D^{⋆}\left(M\right)=\min\left\{D:\exists \left(M,D\right)code\right\}. \end{array}$$

One may consider a stronger criterion that restricts the probability of exceeding a given distortion level.

**Definition** **2** (Excess distortion criterion)**.** *An (M,D,ϵ) code is a pair of an encoder f with |f|≤M and a decoder g such that*

$$\begin{array}{c} \mathrm{Pr}[d(X,g(f(X)))>D]\leq \epsilon . \end{array}$$

*The minimum number of codewords required to achieve excess distortion probability ϵ, and distortion D is defined by*

$$\begin{array}{c} M^{⋆}\left(D,\epsilon \right)=\min\left\{M:\exists \left(M,D,\epsilon \right)code\right\}. \end{array}$$

*Similarly, we can define the minimum achievable excess distortion probability given target distortion D and number of codewords M.*

$$\begin{array}{c} \epsilon^{⋆}\left(M,\epsilon \right)=\min\left\{\epsilon :\exists \left(M,D,\epsilon \right)code\right\}. \end{array}$$

Given target distortion *D* and $p_{X}$, the information rate-distortion function is defined by

(1) $$\begin{array}{c} R\left(D\right)=\underset{p_{\hat{X}|X}:E[d(X,\hat{X})]\leq D}{\inf}I\left(X;\hat{X}\right) \end{array}$$

We make the following benign assumptions:

- There exists a unique rate-distortion function achieving conditional distribution $p_{\hat{X}|X}^{⋆}$.
- We assume that $p_{\hat{X}}^{⋆}\left(\hat{x}\right)>0$ for all $\hat{x}$ ∈ $\hat{X}$ since we can always discard the reconstruction symbol with zero probability.
- If $d\left(x,{\hat{x}}_{1}\right)=d\left(x,{\hat{x}}_{2}\right)$ for all x∈X, then ${\hat{x}}_{1}={\hat{x}}_{2}$. (If $d\left(x,{\hat{x}}_{1}\right)=d\left(x,{\hat{x}}_{2}\right)$ for all *x*, then, there is no difference between ${\hat{x}}_{1}$ and ${\hat{x}}_{2}$ in terms of loss. Thus, we can always discard ${\hat{x}}_{2}$ without loss of generality.)

### 2.3. D-Tilted Information

Define the information density of joint distribution $p_{X,\hat{X}}$ by

$$\begin{array}{c} {𝚤}_{X;\hat{X}}\left(x;\hat{x}\right)=\log\frac{p_{X,\hat{X}}\left(x,\hat{x}\right)}{p_{X}\left(x\right)p_{\hat{X}}\left(\hat{x}\right)}. \end{array}$$

Then, we are ready to define *D*-tilted information that plays a key role in fixed-length lossy compression.

**Definition** **3** ([13] (Definition 6))**.** *The D-tilted information in x∈X is defined as*

$$\begin{array}{c} {𝚥}_{X}\left(x,D\right)=\log\frac{1}{E[\exp\left(\lambda^{⋆}D-\lambda^{⋆}d\left(x,{\hat{X}}^{⋆}\right)\right)]} \end{array}$$

*where the expectation is with respect to the marginal distribution of ${\hat{X}}^{⋆}$ and $\lambda^{⋆}=-R^{\prime }\left(D\right)$.*

Note that ${\hat{X}}^{⋆}$ is a random variable that has a marginal distribution of $p_{X}\times p_{\hat{X}|X}^{⋆}$, and $R^{\prime }\left(D\right)$ is the first derivative of rate-distortion function R(D).

**Theorem** **1** ([14] (Lemma 1.4))**.** *For all $\hat{x}$ ∈ $\hat{X}$,*

(2) $$\begin{array}{c} {𝚥}_{X}\left(x,D\right)={𝚤}_{X;{\hat{X}}^{⋆}}\left(x;\hat{x}\right)+\lambda^{⋆}d\left(x,\hat{x}\right)-\lambda^{⋆}D; \end{array}$$

*therefore, we have*

$$\begin{array}{c} R(D)=E[𝚥(X,D)]. \end{array}$$

Let $p_{X|\hat{X}}^{⋆}$ be the induced conditional probability from $p_{\hat{X}|X}^{⋆}$. Then, (2) can equivalently be expressed as

(3) $$\begin{array}{c} \log\frac{1}{p_{X|\hat{X}}^{⋆}\left(x|\hat{x}\right)}\\ =\log\frac{1}{p_{X}\left(x\right)}-{𝚥}_{X}\left(x,D\right)+\lambda^{⋆}d\left(x,\hat{x}\right)-\lambda^{⋆}D. \end{array}$$

The following lemma shows that $p_{X|\hat{X}}^{⋆}\left(\cdot |\hat{x}\right)$ are all distinct.

**Lemma** **1** ([7] (Lemma 2))**.** *For all ${\hat{x}}_{1}\neq {\hat{x}}_{2}$, there exists x∈X such that $p_{X|\hat{X}}^{⋆}\left(x|{\hat{x}}_{1}\right)\neq p_{X|\hat{X}}^{⋆}\left(x|{\hat{x}}_{2}\right)$.*

## 3. One-to-One Correspondence Between General Distortion and Logarithmic Loss

### 3.1. Main Results

Consider fixed-length lossy compression under arbitrary distortion d(·,·), as described in Section 2.2. We have a source *X* with finite alphabet X={1,…,r}, source distribution $p_{X}$, and finite reconstruction alphabet $\hat{X}=\{1,\ldots ,s\}$. For a fixed number of messages *M*, let $f^{⋆}$ and $g^{⋆}$ be the encoder and decoder that achieve optimal average distortion $D^{⋆}\left(M\right)$, i.e.,

$$\begin{array}{c} E[d(X,g^{⋆}\left(f^{⋆}\left(X\right)\right))]=D^{⋆}\left(M\right). \end{array}$$

Let $p_{\hat{X}|X}^{⋆}$ denote the rate-distortion function achieving conditional distribution at distortion $D=D^{⋆}\left(M\right)$. In other words, $p_{X}\times p_{\hat{X}|X}^{⋆}$ achieves the infimum in

(4) $$\begin{array}{c} R\left(D^{⋆}\left(M\right)\right)=\underset{p_{\hat{X}|X}:E[d(X,\hat{X})]\leq D^{⋆}\left(M\right)}{\inf}I\left(X;\hat{X}\right). \end{array}$$

Note that $R(D^{⋆}\left(M\right))$ may be strictly smaller than logM in general since R(·) is an information rate-distortion function that does not characterize the best achievable performance for the “one-shot” setting in which $D^{⋆}\left(M\right)$ is defined.

Now, we define the corresponding fixed-length lossy-compression problem under logarithmic loss. In the corresponding problem, source alphabet X={1,…,r}, source distribution $p_{X}$, and number of messages *M* remain the same. However, we have a different reconstruction alphabet $Y=\{p_{X|\hat{X}}^{⋆}\left(\cdot |\hat{x}\right):\hat{x}\in \hat{X}\}\subset M(X)$ where $p^{⋆}$ pertains to the achiever of the infimum in Equation (4) associated with the original loss function. Recall that M(X) is the set of all probability measures on 𝒳. Let the distortion of the corresponding problem be the logarithmic loss.

We now further connect the encoding and decoding schemes between the two problems. Suppose f:→X{1,…,M} and $g:\{1,\ldots ,M\}\to \hat{X}$ are an encoder and decoder pair in the original problem. When *f* and *g* are given in the original problem, we define the corresponding encoder and decoder in the corresponding problem as follows. We let the encoder be the same $f_{\ell }=f$, and define the decoder $g_{\ell }:\left\{1,\ldots ,M\right\}\to Y$ by

$$\begin{array}{c} g_{\ell }\left(m\right)=p_{X|\hat{X}}^{⋆}\left(\cdot |g\left(m\right)\right). \end{array}$$

Then, $f_{\ell }$ and $g_{\ell }$ are a valid encoder and decoder pair for the corresponding fixed-length lossy-compression problem under logarithmic loss. Conversely, given $f_{\ell }$ and $g_{\ell }$, we can find corresponding *f* and *g* because Lemma 1 guarantees that $p_{X|\hat{X}}\left(\cdot |\hat{x}\right)$ are distinct.

The following result shows the relation between the corresponding schemes.

**Theorem** **2.** *For any encoder–decoder pair $\left(f_{\ell },g_{\ell }\right)$ for the corresponding fixed-length lossy-compression problem under logarithmic loss, we have*

$$\begin{array}{c} E[\ell (X,g_{\ell }\left(f_{\ell }\left(X\right)\right))]\\ =H\left(X|{\hat{X}}^{⋆}\right)+\lambda^{⋆}\left(E[d(X,g(f(X)))]-D^{⋆}\left(M\right)\right)\\ \geq H(X|{\hat{X}}^{⋆}) \end{array}$$

*where (f,g) is the corresponding encoder–decoder pair for the original lossy-compression problem. Note that $H(X|{\hat{X}}^{⋆})$ and the expectations are with respect to distribution $p_{X}\times p_{\hat{X}|X}^{⋆}$. Moreover, equality holds if and only if $f_{\ell }=f^{⋆}$ and $g_{\ell }\left(m\right)=p_{X|\hat{X}}^{⋆}\left(\cdot |g^{⋆}\left(m\right)\right)$.*

**Proof.**  We have

$$\begin{array}{c} E[\ell (X,g_{\ell }\left(f_{\ell }\left(X\right)\right))]\\ =E[\ell \left(X,p_{X|\hat{X}}^{⋆}\left(\cdot |g\left(f\left(X\right)\right)\right)\right)]\\ =E[\log\frac{1}{p_{X|\hat{X}}^{⋆}\left(X|g\left(f\left(X\right)\right)\right)}]. \end{array}$$

Then, Equation (3) implies that

(5) $$\begin{array}{c} E[\ell (X,g_{\ell }\left(f_{\ell }\left(X\right)\right))]\\ =E[\log\frac{1}{p_{X}\left(X\right)}-{𝚥}_{X}\left(X,D^{⋆}\left(M\right)\right)]\\ \;\;\;+E[\lambda^{⋆}d\left(X,g\left(f\left(X\right)\right)\right)-\lambda^{⋆}D^{⋆}\left(M\right)]\\ =H\left(X|{\hat{X}}^{⋆}\right)+\lambda^{⋆}\left(E[d(X,g(f(X)))-D^{⋆}\left(M\right)\right)] \end{array}$$

(6) $$\begin{array}{c} \geq H(X|{\hat{X}}^{⋆}) \end{array}$$

where Equation (5) is because $E[{}_{𝚥X}\left(X,D^{⋆}\left(M\right)\right)]=R\left(D^{⋆}\left(M\right)\right)=I\left(X;{\hat{X}}^{⋆}\right)$ with respect to distribution $p_{X}\times p_{\hat{X}|X}^{⋆}$. Inequality (6) is because $D^{⋆}\left(M\right)$ is the minimum achievable average distortion with *M* codewords. Equality holds if and only if $E[d(X,g(f(X)))]=D^{⋆}\left(M\right)$, which can be achieved by the optimal scheme for the original lossy-compression problem. In other words, the equality holds if

$$\begin{array}{cc} f_{\ell }^{⋆}= & f^{⋆}\\ g_{\ell }^{⋆}\left(m\right)= & p_{X|\hat{X}}^{⋆}\left(\cdot |g^{⋆}\left(m\right)\right). \end{array}$$

 □

In the above theorem, distortion $D^{⋆}\left(M\right)$ plays a critical role, which is the minimal achievable distortion in the one-shot setting. We also used $p_{X|\hat{X}}^{⋆}$ in the corresponding problem, which is the rate-distortion-achieving conditional distribution. This might be confusing since the rate-distortion function provides the optimal rate in the asymptotic setting. However, recall that the minimal mutual information between *X* and $\hat{X}$ in Equation (1) is the “information” rate-distortion function. The “information” rate-distortion function is equal to the optimum rate in the asymptotic case if the source is independent and identically distributed.

On the other hand, we viewed the “information” rate-distortion function differently. We considered the one-shot setting where source *X* and reconstruction $\hat{X}$ are single variables. Given number of messages *M*, the minimal achievable distortion is given by $D^{⋆}\left(M\right)$. Under this setting, we focused on minimal mutual information between *X* and $\hat{X}$ when the distortion between *X* and $\hat{X}$ is restricted by $D^{⋆}\left(M\right)$. Our theorem implies that minimal achieving distribution $p_{X|\hat{X}}^{⋆}$ provides the corresponding one-shot lossy-compression problem under logarithmic loss.

**Remark** **1.** *In the corresponding fixed-length lossy-compression problem under logarithmic loss, the minimal achievable average distortion given number of codewords M is*

$$\begin{array}{c} D_{\ell }^{⋆}\left(M\right)=H\left(X|{\hat{X}}^{⋆}\right) \end{array}$$

*where the conditional entropy is with respect to distribution $p_{X}\times p_{\hat{X}|X}^{⋆}$.*

**Remark** **2.** *From now on, we denote the original lossy-compression problem under given distortion measure d(·,·) with reconstruction alphabet $\hat{X}$ by “original problem”. On the other hand, we denote the corresponding lossy-compression problem under logarithmic loss with reconstruction alphabet Y by “corresponding problem”.*

### 3.2. Example: Memoryless Bernoulli Source with Hamming Distortion Measure

In this section, we consider the memoryless Bernoulli source under Hamming distortion measure as an example of the above equivalence. Let $X=U^{n}$ be a memoryless Bernoulli source with probability α, where $X=U^{n}={\left\{0,1\right\}}^{n}$, and reconstruction $\hat{X}=V^{n}$ is also an *n*-dimensional binary vector where $\hat{X}=V^{n}={\left\{0,1\right\}}^{n}$. Note that block length *n* is fixed, so the problem is in the one-shot setting. Distortion measure *d* is separable Hamming distortion, i.e.,

$$\begin{array}{c} d\left(X,\hat{X}\right)=d_{H}\left(U^{n},V^{n}\right)=\frac{1}{n}\sum_{i=1}^{n}d_{H}\left(U_{i},V_{i}\right) \end{array}$$

where $d_{H}\left(u,v\right)=1$ if u≠v and $d_{H}\left(u,v\right)=0$ if u=v. Let *M* be the number of messages. Then, we are interested in optimal encoding and decoding schemes that achieve distortion $D=D^{⋆}\left(M\right)$.

In this scenario, the information rate-distortion function is not hard to compute [15]:

(7) $$\begin{array}{cc} R(D)= & \underset{p_{\hat{X}|X}:E[d(X,\hat{X})]\leq D}{\inf}I\left(X;\hat{X}\right)\\ = & \underset{p_{U^{n}|V^{n}}:E[d_{H}\left(U^{n},V^{n}\right)]\leq D}{\inf}I\left(U^{n};V^{n}\right)\\ = & n\underset{p_{U|V}:E[d_{H}\left(U,V\right)]\leq D}{\inf}I\left(U;V\right) \end{array}$$

(8) $$\begin{array}{cc} = & n(h_{2}\left(\alpha \right)-h_{2}\left(D\right)), \end{array}$$

where $h_{2}\left(\cdot \right)$ is the binary entropy function. Let $p_{U|V}^{⋆}$ be the distribution that achieves the infimum in Equation (7). We have an analytic formula for rate-distortion-achieving distribution $p_{X|\hat{X}}^{⋆}$. For $x=u^{n}$ and $=v^{n}$, we have

$$\begin{array}{cc} p_{X|\hat{X}}^{⋆}\left(x|\hat{x}\right)= & \prod_{i=1}^{n}p_{U|V}^{⋆}\left(u_{i}|v_{i}\right)\\ = & \prod_{i=1}^{n}D^{d_{H}\left(u_{i},v_{i}\right)}{\left(1-D\right)}^{1-d_{H}\left(u_{i},v_{i}\right)}\\ = & {\left(1-D\right)}^{n}{\left(\frac{D}{1-D}\right)}^{nd_{H}\left(u^{n},v^{n}\right)}\\ = & {\left(1-D\right)}^{n}{\left(\frac{D}{1-D}\right)}^{nd(x,\hat{x})}. \end{array}$$

Then, the corresponding problem is the rate-distortion problem under logarithmic loss where the set of reconstruction symbols is

$$\begin{array}{cc} Y= & \{p_{X|\hat{X}}^{⋆}\left(\cdot |\hat{x}\right):\hat{x}\in V^{n}\}. \end{array}$$

**Remark** **3.** *We can rewrite Equation (3) in this case.*

$$\begin{array}{cc} \ell (x,p_{X|\hat{X}}^{⋆}\left(\cdot |\hat{x}\right))= & \log\frac{1}{p_{X|\hat{X}}^{⋆}\left(x|\hat{x}\right)}\\ = & n\log\frac{1}{1-D}+nd\left(x,\hat{x}\right)\log\frac{1-D}{D}. \end{array}$$

*The above equation explicitly shows the correspondence between logarithmic loss and the original distortion measure.*

### 3.3. Discussion

#### 3.3.1. One-to-One Correspondence

Theorem 2 implies that, for any fixed-length lossy-compression problem, we can find an equivalent problem under logarithmic loss where optimal encoding schemes are the same. Thus, without loss of generality, we can restrict our attention to the problem under logarithmic loss with reconstruction alphabet $Y=\{q^{\left(1\right)},\ldots ,q^{\left(s\right)}\}$ for some $q^{\left(1\right)},\ldots ,q^{\left(s\right)}\in M\left(X\right)$.

#### 3.3.2. Scheme Suboptimality

Suppose *f* and *g* are a suboptimal encoder and decoder for the original fixed-length lossy-compression problem. Then, the theorem implies

(9) $$\begin{array}{c} E[\ell (X,g_{\ell }\left(X\right))-H\left(X|{\hat{X}}^{⋆}\right)]\\ =\lambda^{⋆}\left(E[d(X,g(f(X)))]-D^{⋆}\left(M\right)\right). \end{array}$$

The left-hand side of Equation (9) is the cost of suboptimality for the corresponding lossy-compression problem. On the other hand, the right-hand side is proportional to the cost of suboptimality for the original problem. In Section 3.3.1, we discussed that the optimal schemes of the two problems coincide. Equation (9) shows stronger equivalence in which costs of suboptimalities are linearly related. This implies that a good code for one problem is also good for the other.

#### 3.3.3. Operations on the Reconstruction Alphabet

In general, reconstruction alphabet $\hat{X}$ does not have an algebraic structure. However, in the corresponding rate-distortion problem, the reconstruction alphabet is the set of probability measures where we have natural operations such as convex combinations of elements or projection to a convex hull. We discuss such operations closer in Section 5.

### 3.4. Exact Performance of Optimal Scheme

In the previous section, we showed that there is a corresponding lossy-compression problem under logarithmic loss that shares the same optimal coding scheme. In this section, we investigate the exact performance of the optimal scheme for the fixed-length lossy-compression problem under logarithmic loss, when the reconstruction alphabet is the set of all probability measures on 𝒳, i.e., M(X). (Recently, Shkel and Verdu [4] independently proposed similar results. The result was also presented in our conference version of the paper [16].) We also characterize minimal average distortion $D^{⋆}\left(M\right)$ when we have a fixed number of messages *M*. Note that this is a single-letter version of ([2], [Lemma 1]). Although the optimal scheme associated with M(X) may differ from the optimal scheme with restricted reconstruction alphabets Y, it provides an insight, as we show in Section 4. In this section, we restrict our attention to deterministic schemes. However, it is not hard to show that the same result holds even if we allow a stochastic encoder and decoder.

Let an encoder and a decoder be f:X→{1,…,M} and g:{1,…,M}→M(X) where $g\left(m\right)=q^{\left(m\right)}\in M(X)$. Then, we have

$$\begin{array}{c} E[\ell (X,g(f(X)))]\\ =\sum_{x\in X}p_{X}\left(x\right)\log\frac{1}{q^{\left(f(x)\right)}\left(x\right)}\\ =H\left(X\right)+\sum_{m=1}^{M}\sum_{x\in f^{-1}\left(m\right)}p_{X}\left(x\right)\log\frac{p_{X}\left(x\right)}{q^{\left(m\right)}\left(x\right)}\\ =H\left(X\right)+\sum_{m=1}^{M}u_{m}\log u_{m}\\ \;\;\;+\sum_{m=1}^{M}u_{m}\sum_{x\in f^{-1}\left(m\right)}\frac{p_{X}\left(x\right)}{u_{m}}\log\frac{p_{X}\left(x\right)/u_{m}}{q^{\left(m\right)}\left(x\right)}, \end{array}$$

where $f^{-1}\left(m\right)=\left\{x\in X:f\left(x\right)=m\right\}$ and $u_{m}=\sum_{x\in f^{-1}\left(m\right)}p_{X}\left(x\right)$. Since $p_{X|f(X)}\left(x|m\right)=\frac{p_{X}\left(x\right)}{u_{m}}$ for all $x\in f^{-1}\left(m\right)$, we have

$$\begin{array}{c} E[\ell (X,g(f(X)))]\\ =H(X)-H(f(X))\\ \;\;\;+\sum_{m=1}^{M}u_{m}D\left(p_{X|f(X)}\left(\cdot |m\right)\|q^{\left(m\right)}\right)\\ \geq H(X)-H(f(X)). \end{array}$$

Equality can be achieved by choosing $q^{\left(m\right)}=p_{X|f(X)}\left(\cdot |m\right)$, which can be done no matter what *f* is. Thus, we have

$$\begin{array}{c} D^{⋆}\left(M\right)=H\left(X\right)-\max_{f:|f|\leq M}H\left(f\left(X\right)\right). \end{array}$$

This implies that the optimal encoder is function *f* that maximizes H(f(X)), and the optimal decoder is given by $g\left(m\right)=p_{X|f(X)}\left(\cdot |m\right)$. The above result provides a trivial lower bound:

$$\begin{array}{c} D^{⋆}\left(M\right)\geq H\left(X\right)-\log M. \end{array}$$

The optimal scheme under an excess distortion criterion is given in Appendix A.

## 4. Geometrical Interpretation

In this section, we present another geometrical interpretation of the decoder in lossy-compression problems. Consider the original lossy-compression problem with discrete reconstruction alphabet $\hat{X}$ and distortion measure d(·,·). Suppose encoding function *f* is given that may or may not be optimal, where |f|=M. Let $A_{m}=f^{-1}\left(m\right)=\left\{x\in X:f\left(x\right)=m\right\}$, which is the set of source symbols that are mapped to message *m*. Then, optimal reconstruction g(m) is given by

(10) $$\begin{array}{c} g\left(m\right)=\underset{\hat{x}\in \hat{X}}{\mathrm{argmin}}E[d\left(X,\hat{x}\right)|X\in A_{m}]. \end{array}$$

Now, consider the corresponding lossy-compression problem under logarithmic loss. Recall that the set of reconstruction alphabets is given by

$$\begin{array}{c} Y=\{p_{X|\hat{X}}^{⋆}\left(\cdot |\hat{x}\right):\hat{x}\in \hat{X}\} \end{array}$$

where Y⊂M(X). As we have seen in Section 3.4, the optimal reconstruction is $g_{\ell }^{E}\left(m\right)=p_{X|f(X)}\left(\cdot |m\right)$ if we have extended set of reconstruction alphabet M(X). Thus, it is natural to find the probability distribution in 𝒴, which is the nearest distribution from $g_{\ell }^{E}\left(m\right)$. We propose Kullback–Leibler divergence to measure the distance between probability distributions. In other words, we want to find ${\tilde{g}}_{\ell }\left(m\right)\in Y$, such that

(11) $$\begin{array}{c} {\tilde{g}}_{\ell }\left(m\right)=\underset{q\in Y}{\mathrm{argmin}}D\left(g_{\ell }^{E}\left(m\right)∥q\right). \end{array}$$

This can be viewed as projecting the optimal solution from extended set M(X) to original feasible set 𝒴. Since q∈Y, there exists $\hat{x}$ ∈ $\hat{X}$, such that $q\left(\cdot \right)=p_{X|\hat{X}}^{⋆}\left(\cdot |\hat{x}\right)$. Then, the above Kullback–Leibler divergence is given by

$$\begin{array}{c} D(p_{X|f(X)}\left(\cdot |m\right)∥p_{X|\hat{X}}^{⋆}\left(\cdot |\hat{x}\right))\\ =\sum_{x\in A_{m}}p_{X|f(X)}\left(x|m\right)\log\frac{p_{X|f(X)}\left(x|m\right)}{p_{X|\hat{X}}^{⋆}\left(x|\hat{x}\right)}\\ =\sum_{x\in A_{m}}\frac{p_{X}\left(x\right)}{\mathrm{Pr}[X\in A_{m}]}\log\frac{p_{X}\left(x\right)}{\mathrm{Pr}[X\in A_{m}]p_{X|\hat{X}}^{⋆}\left(x|\hat{x}\right)}\\ =\log\frac{1}{\mathrm{Pr}[X\in A_{m}]}+\sum_{x\in A_{m}}\frac{p_{X}\left(x\right)}{\mathrm{Pr}[X\in A_{m}]}\log\frac{p_{X}\left(x\right)}{p_{X|\hat{X}}^{⋆}\left(x|\hat{x}\right)}\\ =\log\frac{1}{\mathrm{Pr}[X\in A_{m}]}+\sum_{x\in A_{m}}\frac{p_{X}\left(x\right)}{\mathrm{Pr}[X\in A_{m}]}\left(-𝚥\left(x,D\right)+\lambda^{⋆}d\left(x,\hat{x}\right)-\lambda^{⋆}D\right), \end{array}$$

where the last equality is from Equation (2). Note that $d(x,\hat{x})$ is the only term that is a function of $\hat{x}$, and $\lambda^{⋆}$ is positive. Thus, if $q\left(\cdot \right)=p_{X|\hat{X}}\left(\cdot |\hat{x}\right)$ achieves the minimum in Equation (11), then $\hat{x}$ minimizes the following:

(12) $$\begin{array}{c} \sum_{x\in A_{m}}\frac{p_{X}\left(x\right)}{\mathrm{Pr}[X\in A_{m}]}d\left(x,\hat{x}\right)=E[d\left(X,\hat{x}\right)|X\in A_{m}]. \end{array}$$

Since Equation (12) coincides with Equation (10), we have

(13) $$\begin{array}{c} {\tilde{g}}_{\ell }\left(m\right)=p_{X|\hat{X}}^{⋆}\left(\cdot |g\left(m\right)\right). \end{array}$$

**Remark** **4.** *In Section 3, we directly defined $g_{\ell }\left(m\right)=p_{X|\hat{X}}^{⋆}\left(\cdot |g\left(m\right)\right)$. However, we obtained ${\tilde{g}}_{\ell }\left(m\right)$ via the following two-step procedure:*

- *extend the reconstruction set from 𝒴 to M(X), then characterize optimal decoding functions $g_{\ell }^{E}\left(m\right)\in M(X)$; and*
- *find the measure ${\tilde{g}}_{\ell }\left(m\right)\in Y$ that is closest to $g_{\ell }^{E}\left(m\right)$.*

*The above result (13) implies that ${\tilde{g}}_{\ell }\left(m\right)=g_{\ell }\left(m\right)$.*

## 5. Log-Convex Relaxation

In the previous section, we obtained the optimal reconstruction symbol from the extended reconstruction alphabet, and projected it to the feasible set. In this section, instead of direct projection to 𝒴, we propose another slight extension of 𝒴, namely, log-convex hull. As we show in the following sections, the log-convex hull has interesting properties.

### 5.1. rI-Projection

Before defining the log-convex hull, we need to define the log-convex combination of probability distributions. Let *p* and *q* be probability distributions in M(X). For 0<t<1, the log-convex combination of *p* and *q* is given by

(14) $$\begin{array}{c} \bar{p^{t}q^{1-t}}\left(x\right)=\frac{p{\left(x\right)}^{t}q{\left(x\right)}^{1-t}}{\sum_{\tilde{x}}p{\left(\tilde{x}\right)}^{t}q{\left(\tilde{x}\right)}^{1-t}}. \end{array}$$

It is clear to see that $\log\bar{p^{t}q^{1-t}}$ is a convex combination of logp(x) and logq(x) with a normalizing constant. We can now define log-convex hull logconv(Y) that is a set of log-convex combination of probability measures in set 𝒴. More precisely,

$$\begin{array}{c} \mathrm{logconv}(Y)=\left\{q^{\left(r\right)}\in M\left(X\right):q^{\left(r\right)}\left(x\right)=\frac{1}{c(r)}\exp\left(\sum_{\hat{x}}r\left(\hat{x}\right)\log p_{X|\hat{X}}^{⋆}\left(x|\hat{x}\right)\right)\right\} \end{array}$$

where *r* is a weight vector (i.e., r∈M(X)), and c(r) is a normalizing constant. By definition, logconv(Y) is log-convex since it contains all log-convex combinations of probability distributions in 𝒴.

Instead of having projection of $p_{X|f(X)}\left(\cdot |m\right)$ to 𝒴, we consider the projection to logconv(Y). Since logconv(Y) is log-convex, ([17], [Theorem 1]) implies that there exists unique probability distribution $q_{m}^{⋆}\in \mathrm{logconv}(Y)$ that achieves the following minimum.

$$\begin{array}{c} \min_{q\in \mathrm{logconv}(Y)}D\left(p_{X|f(X)}\left(\cdot |m\right)∥q\right). \end{array}$$

Projection $q_{m}^{⋆}$ is called an *rI*-projection of $p_{X|f(X)}\left(\cdot |m\right)$ to logconv(Y). Let $r_{m}^{⋆}$ be the corresponding weights, i.e.,

$$\begin{array}{c} q_{m}^{⋆}=q^{\left(r_{m}^{⋆}\right)}. \end{array}$$

Csiszár and Matúš ([17], [Theorem 1]) showed that the *rI*-projection satisfies the following inequality for all $\hat{x}$ ∈ $\hat{X}$.

(15) $$\begin{array}{c} D\left(p_{X|f(X)}\left(\cdot |m\right)∥p_{X|\hat{X}}^{⋆}\left(\cdot |\hat{x}\right)\right)\geq D\left(p_{X|f(X)}\left(\cdot |m\right)∥q_{m}^{⋆}\right)+D\left(q_{m}^{⋆}∥p_{X|\hat{X}}^{⋆}\left(\cdot |\hat{x}\right)\right). \end{array}$$

On the other hand, the log-convex combination of probability measures $q^{\left(r\right)}$ is called the geometric mean of probability measures [18]. The author also provided geometric compensation identity, which is given by

(16) $$\begin{array}{cc} \sum_{\hat{x}}r\left(\hat{x}\right)D\left(p_{X|f(X)}\left(\cdot |m\right)∥p_{X|\hat{X}}^{⋆}\left(\cdot |\hat{x}\right)\right)= & D\left(p_{X|f(X)}\left(\cdot |m\right)∥q^{\left(r\right)}\right)+\sum_{\hat{x}}r\left(\hat{x}\right)D\left(q^{\left(r\right)}∥p_{X|\hat{X}}^{⋆}\left(\cdot |\hat{x}\right)\right). \end{array}$$

The above result holds for any r∈M(X); therefore, Equation (16) also holds when $q^{\left(r\right)}=q_{m}^{*}$. Together with Inequality (15), we get the following result. For all $\hat{x}$ ∈ $\hat{X}$, if $r_{m}^{⋆}\left(\hat{x}\right)\neq 0$, then

$$\begin{array}{cc} D(p_{X|f(X)}\left(\cdot |m\right)∥p_{X|\hat{X}}^{⋆}\left(\cdot |\hat{x}\right))= & D\left(p_{X|f(X)}\left(\cdot |m\right)∥q_{m}^{⋆}\right)+D\left(q_{m}^{⋆}∥p_{X|\hat{X}}^{⋆}\left(\cdot |\hat{x}\right)\right). \end{array}$$

**Remark** **5.** *The above result is similar to the projection to polytope in Euclidean space. Suppose vectors $v_{1},v_{2},\ldots ,v_{n}$ form a polytope, and consider the projection from a vector w to the polytope. Let h be a projection. Then, h is a convex combination of $v_{i}$’s. Thus, there exist coefficients ${\left\{a_{i}\right\}}_{1\leq i\leq n}$, such that*

$$\begin{array}{c} h=\sum_{i=1}^{n}a_{i}v_{i} \end{array}$$

*where $\sum_{i}a_{i}=1$, and $a_{i}\geq 0$ for all i. Let $E=\{1\leq i\leq n:a_{i}\neq 0\}$ be the set of indices of nonzero coefficients. Then, projection h is on the plane generated by ${\left\{v_{i}\right\}}_{i\in E}$. Thus, two vectors w−h and $h-v_{i}$ are orthogonal for all i∈E. Then, Pythagorean theorem implies that, for all i, we have either $a_{i}=0$ or*

$$\begin{array}{c} ∥w-v_{i}{∥}^{2}={∥w-h∥}^{2}+{∥h-v_{i}∥}^{2}. \end{array}$$

### 5.2. Optimization

As we saw in the previous section, we want to find q∈logconv(Y) that minimizes $D\left(p_{X|f(X)}\left(\cdot |m\right)∥q\right)$. Note that

$$\begin{array}{cc} D\left(p_{X|f(X)}\left(\cdot |m\right)∥q^{\left(r\right)}\right)= & \sum_{x\in X}p_{X|f(X)}\left(x|m\right)\log\frac{p_{X|f(X)}\left(x|m\right)}{q^{\left(r\right)}\left(x\right)}\\ = & \sum_{x\in X}p_{X|f(X)}\left(x|m\right)\log p_{X|f(X)}\left(x|m\right)+\sum_{x\in X}p_{X|f(X)}\left(x|m\right)\log\frac{1}{q^{\left(r\right)}\left(x\right)}. \end{array}$$

Since the first term is not a function of $q^{\left(r\right)}$, it is enough to consider the second term. By the definition of $q^{\left(r\right)}$, we have

$$\begin{array}{cc} \sum_{x\in X}p_{X|f(X)}\left(x|m\right)\log\frac{1}{q^{\left(r\right)}\left(x\right)}= & -\sum_{x\in X}p_{X|f(X)}\left(x|m\right)\sum_{\hat{x}}r\left(\hat{x}\right)\log p_{X|\hat{X}}^{⋆}\left(x|\hat{x}\right)+\log c\left(r\right)\\ = & -\sum_{x\in X}p_{X|f(X)}\left(x|m\right)\sum_{\hat{x}}r\left(\hat{x}\right)\log p_{X|\hat{X}}^{⋆}\left(x|\hat{x}\right)\\ & +\log\left(\sum_{x^{\prime }}\exp\left(\sum_{\hat{x}}r\left(\hat{x}\right)\log p_{X|\hat{X}}^{⋆}\left(x^{\prime }|\hat{x}\right)\right)\right). \end{array}$$

Thus, minimizing $D(p_{X|f(X)}\left(\cdot |m\right)∥q)$ is equivalent to solving the following optimization problem.

$$\begin{array}{cc} \min_{r\in M(\hat{X})}\; & \;-\sum_{\hat{x}}r\left(\hat{x}\right)\sum_{x}p_{X|f(X)}\left(x|m\right)\log p_{X|\hat{X}}^{⋆}\left(x|\hat{x}\right)+\log\left(\sum_{x^{\prime }}\exp\left(\sum_{\hat{x}}r\left(\hat{x}\right)\log p_{X|\hat{X}}^{⋆}\left(x^{\prime }|\hat{x}\right)\right)\right)\\ s.t.\; & \;r(\hat{x})\geq 0\\ \; & \;\sum_{\hat{x}}r\left(\hat{x}\right)=1. \end{array}$$

Since the objective function is a convex function of $r(\hat{x})$, the above problem is a convex optimization problem that can be efficiently solved.

### 5.3. Relaxation in Clustering

In the corresponding lossy-compression problem under logarithmic loss, reconstruction symbols are probability measures that have a natural algebraic structure, as we discussed in Section 3.3.3. In this section, we present the benefits of such a property when we apply some known techniques from the clustering literature.

Lossy compression is closely related to the clustering problem [19,20,21]. Many works focused on the application of *k*-means clustering to a lossy-compression problem [22,23,24], which is an extension of the Lloyd max algorithm [25,26]. However, *k*-means clustering is only available when there exists a well-defined operation in $\hat{X}$ (e.g., $\hat{X}=R^{n}$). This is because *k*-means clustering requires computing the mean of data points, which is the center of each cluster. In general lossy-compression problems, reconstruction alphabet $\hat{X}$ may not have such an operation. In such cases, we may have to apply *k*-medoidlike clustering [27], where the center of each cluster has to be a data point. The *k*-medoidlike algorithm in the context of lossy compression is shown in Algorithm 1.

**Algorithm 1***k*-medoidlike clustering in lossy compression. Randomly initialize ${\hat{x}}_{1},\ldots ,{\hat{x}}_{M}\in \hat{X}$ **repeat**  Set $A_{m}\leftarrow \emptyset$ for all 1≤m≤M.  **for**
x∈X
**do**   $A_{m}\leftarrow A_{m}\cup \left\{x\right\}$ where $m=\underset{m^{\prime }}{\mathrm{argmin}}d\left(x,{\hat{x}}_{m^{\prime }}\right)$  **end for**  **for**
m=1 to *M*
**do**   ${\hat{x}}_{m}\leftarrow \underset{\hat{x}\in \hat{X}}{\mathrm{argmin}}\sum_{x\in A_{m}}p_{X}\left(x\right)d\left(x,\hat{x}\right)$  **end for** **until** converge

On the other hand, in the corresponding problem, the reconstruction alphabet is the set of probability distributions where operations such as log-convex combinations are well-defined. This allows us to propose a *k*-meanslike clustering algorithm, as shown in Algorithm 2.

**Algorithm 2***k*-meanslike clustering in lossy compression. Randomly initialize $r_{1},\ldots ,r_{M}\in M(\hat{X})$ **repeat**  Set $A_{m}\leftarrow \emptyset$ for all 1≤m≤M  **for**
x∈X
**do**   $A_{m}\leftarrow A_{m}\cup \left\{x\right\}$ where $m=\underset{m^{\prime }}{\mathrm{argmin}}\log\frac{1}{q^{\left(r_{m^{\prime }}\right)}\left(x\right)}$   Set f(x)=m where $x\in A_{m}$  **end for**  **for**
m=1 to *M*
**do**   $r_{m}\leftarrow \underset{r\in M(\hat{X})}{\mathrm{argmin}}D\left(p_{X|f(X)}\left(\cdot |m\right)∥q^{\left(r\right)}\right)$  **end for** **until** converge

The main idea of the above algorithm is that log-convex combination $q_{m}^{⋆}$ behaves like center of cluster $A_{m}$. In the clustering literature, there are many known variations of *k*-means clustering [28,29]. The above result shows that we can borrow those techniques and apply them to the lossy-compression problem even without any algebraic structures on the reconstruction alphabet.

### 5.4. Application to General Clustering Problems

The idea of the previous section can be applied to an actual clustering problem. We mainly focus on clustering categorical data where data points are not in continuous space [30,31,32,33,34]. Since operations such as *mean* are not well-defined in this case, it is hard to apply known data-clustering algorithms in continuous space. The key idea is that the equivalence relation with logarithmic loss allows the algebraic structure on any set. More precisely, we can transform any clustering problem to the clustering problem in continuous space and apply known techniques such as variations of *k*-means.

A more rigorous definition of the problem is given below. Assume that we have a finite set of data points 𝒳, and each data point has its weight $p_{X}\left(x\right)$. We normalize the weights so that $\sum_{x}p_{X}\left(x\right)=1$, and the weights may or may not be uniform. The distance between two points are given by measure d:X×X→[0,∞). Suppose we want to partition the data points into *M* clusters.

If we let $\hat{X}$ = 𝒳, then the clustering problem turns out to be a lossy-compression problem under distortion measure d(·,·), where the number of messages is *M*. Let $D=D^{⋆}\left(M\right)$ be the optimal achievable distortion, and $p_{\hat{X}|X}^{⋆}$ be the distribution that achieves rate-distortion function R(D) as defined in Equation (4). Then, we can find the corresponding lossy-compression problem under logarithmic loss. Finally, we can apply clustering algorithms in continuous space such as *k*-means to the corresponding problem. For example, Algorithm 2 can be applied to the corresponding problem.

**Remark** **6.** *Note that it is hard to have an exact analytic formula for $D^{⋆}\left(M\right)$ or $p_{|X\hat{X}}^{⋆}$. However, as we mentioned in Section 3.3.2, we do not have to find an optimal scheme under the exact problem formulation. If we can provide a good scheme of the corresponding problem with $D\approx D^{⋆}\left(M\right)$, that should be a good enough scheme in the original problem.*

## 6. Conclusions

To conclude our discussion, we summarize our main contributions. We showed that for any fixed-length lossy-compression problem under an arbitrary distortion measure, there exists a corresponding lossy-compression problem under logarithmic loss where optimal schemes coincide. We also proved that a good scheme for one lossy-compression problem is also good for another problem. This equivalence provides an algebraic structure on any reconstruction alphabet that allows using various optimization techniques in lossy-compression problems, such as log-convex relaxation. Furthermore, our results naturally suggest a *k*-meanslike clustering algorithm in categorical data-clustering problems.

## Appendix A. Optimal Scheme Under Excess Distortion Criterion

In this section, we characterize minimum number of codewords $M^{⋆}\left(D,\epsilon \right)$ that can achieve distortion *D* and excess distortion probability ϵ. Let an encoder and a decoder be f:X→{1,…,M} and g:{1,…,M}→M(X) where $g\left(m\right)=q^{\left(m\right)}\in M(X)$. Since ℓ(x,q)≤D is equivalent to $q\left(x\right)\geq e^{-D}$, we hav

$$\begin{array}{cc} 1-p_{e}= & \sum_{x\in X}p_{X}\left(x\right)1\left(q^{\left(f(x)\right)}\left(x\right)\geq e^{-D}\right)\\ = & \sum_{m=1}^{M}\sum_{x\in f^{-1}\left(m\right)}p_{X}\left(x\right)1\left(q^{\left(m\right)}\left(x\right)\geq e^{-D}\right). \end{array}$$

However, at most, $\left\lfloor e^{D}\right\rfloor$ of the $q^{\left(m\right)}\left(x\right)$ can be larger than $e^{-D}$ where ⌊x⌋ is the largest integer that is smaller than or equal to *x*. Thus, we can at mos cover t $M\cdot \lfloor e^{D}\rfloor$ of the source symbols with *M* codewords. Suppose $p_{X}\left(1\right)\geq p_{X}\left(2\right)\geq \cdots \geq p_{X}\left(r\right)$, then the optimal scheme is

$$\begin{array}{cc} f(x)= & \left\lceil \frac{x}{\left\lfloor e^{D}\right\rfloor }\right\rceil \\ q^{\left(m\right)}\left(x\right)= & \left\{\begin{array}{cc} 1/\lfloor e^{D}\rfloor & \mathrm{if}f(x)=m\\ 0 & \mathrm{otherwise}, \end{array}\right. \end{array}$$

where $q^{\left(m\right)}=g\left(m\right)$ and ⌈x⌉ are the smallest integer that is larger than or equal to *x*. The idea is that each reconstruction symbol $q^{\left(m\right)}$ covers $\left\lfloor e^{D}\right\rfloor$ number of source symbols by assigning probability mass $1/\lfloor e^{D}\rfloor$ to each of them.

The above optimal scheme satisfies

$$\begin{array}{cc} 1-p_{e}= & \sum_{x=1}^{M\cdot \lfloor e^{D}\rfloor }p_{X}\left(x\right)\\ = & F_{X}\left(M\cdot \lfloor e^{D}\rfloor \right), \end{array}$$

where $F_{X}\left(\cdot \right)$ is the cumulative distribution function of *X*. This implies that the minimal error probability is

$$\begin{array}{c} \epsilon^{⋆}\left(M,D\right)=1-F_{X}\left(M\cdot \lfloor e^{D}\rfloor \right). \end{array}$$

On the other hand, if we fix target error probability ϵ, the minimal number of codewords is

$$\begin{array}{c} M^{⋆}\left(D,\epsilon \right)=\left\lceil \frac{F_{X}^{-1}\left(1-\epsilon \right)}{\left\lfloor e^{D}\right\rfloor }\right\rceil \end{array}$$

where $F_{X}^{-1}\left(y\right)=\underset{1\leq x\leq r}{\mathrm{argmin}}\left\{x:F_{X}\left(x\right)\geq y\right\}$. Note that if we allow variable length coding without a prefix condition, the optimal coding scheme is similar to optimal nonasymptotic lossless coding introduced in [35].
